# The involvement of the noradrenergic system in the antinociceptive effect of cucurbitacin D on mice with paclitaxel-induced neuropathic pain

**DOI:** 10.3389/fphar.2022.1055264

**Published:** 2023-01-04

**Authors:** Keun-Tae Park, Suyong Kim, Ilseob Choi, Ik-Hwan Han, Hyunsu Bae, Woojin Kim

**Affiliations:** ^1^ Department of Physiology, College of Korean Medicine, Kyung Hee University, Seoul, South Korea; ^2^ Korean Medicine-Based Drug Repositioning Cancer Research Center, College of Korean Medicine, Kyung Hee University, Seoul, South Korea

**Keywords:** allodynia, chemotherapy-induced neuropathic pain, cucurbitacin B, cucurbitacin D, noradrenergic system, paclitaxel

## Abstract

Paclitaxel (sold under the brand name Taxol) is a chemotherapeutic drug that is widely used to treat cancer. However, it can also induce peripheral neuropathy, which limits its use. Although several drugs are used to attenuate neuropathy, no optimal treatment is available to date. In this study, the effect of cucurbitacins B and D on paclitaxel-induced neuropathic pain was assessed. Multiple paclitaxel injections (a cumulative dose of 8 mg/kg, i. p.) induced cold and mechanical allodynia from days 10 to 21 in mice, and the i. p. administration of 0.025 mg/kg of cucurbitacins B and D attenuated both allodynia types. However, as cucurbitacin B showed a more toxic effect on non-cancerous (RAW 264.7) cells, further experiments were conducted with cucurbitacin D. The cucurbitacin D dose-dependently (0.025, 0.1, and 0.5 mg/kg) attenuated both allodynia types. In the spinal cord, paclitaxel injection increased the gene expression of noradrenergic (*α*_1_-and *α*_2_-adrenergic) receptors but not serotonergic (5-HT_1A_ and _3_) receptors. Cucurbitacin D treatment significantly decreased the spinal *α*_1_- but not *α*_2_-adrenergic receptors, and the amount of spinal noradrenaline was also downregulated. However, the tyrosine hydroxylase expression measured *via* liquid chromatography in the locus coeruleus did not decrease significantly. Finally, cucurbitacin D treatment did not lower the anticancer effect of chemotherapeutic drugs when co-administered with paclitaxel in CT-26 cell-implanted mice. Altogether, these results suggest that cucurbitacin D could be considered a treatment option against paclitaxel-induced neuropathic pain.

## 1 Introduction

Paclitaxel (sold under the brand name Taxol) is a chemotherapeutic drug widely used against tumors, such as ovarian and breast cancers (Rowinsky and Donehower, 1995; Bernabeu et al., 2017). However, along with its anti-tumor effect, paclitaxel can induce neurotoxicity (Rowinsky et al., 1993). Peripheral neuropathy, which is characterized by sensory symptoms such as numbness and paresthesia, is one of the most common and serious adverse effects experienced by paclitaxel-treated patients (Chaudhry et al., 1994; Kosturakis et al., 2014). It can induce both acute and chronic pain in up to 78% of treated patients (Beijers et al., 2012). Paclitaxel is a cytoskeletal drug as it targets tubulins. Its main mechanism of action in cells is to cause a mitotic block by stabilizing the mitotic spindle microtubules, thereby inhibiting the dynamic nature of these cytoskeletal structures (Jordan, 2002). These aggregated microtubules in cells mostly affect the sensory nervous system, while the motor and autonomic nervous systems are less affected (Kuroi and Shimozuma, 2004; Stillman and Cata, 2006; Pachman et al., 2011). Antidepressants are considered the first drug treatment of choice (Dworkin et al., 2007; Finnerup et al., 2015). Among the various types of antidepressants, serotonin norepinephrine reuptake inhibitors (SNRIs) are known to be effective (Durand et al., 2012; Farshchian et al., 2018). Therefore, modulating the serotoninergic and noradrenergic systems in paclitaxel-induced neuropathic pain could be an effective strategy to alleviate cold and mechanical allodynia.

*Trichosanthes kirilowii* Maximowicz (TK) is categorized in the genus *Trichosanthes* and family Cucurbitaceae. Various types of cucurbitacins are largely present in the root tubers of TK. Cucurbitacins are a group of natural triterpenoids and have long been used in traditional medicine (Ríos et al., 2005; Chen et al., 2008; Kaushik et al., 2015). Plants containing cucurbitacins have been reported to have anti-tumor, anti-inflammatory, and anti-bacterial activities (Jayaprakasam et al., 2003; Chen et al., 2005). A total of 17 major molecules from cucurbitacin A to cucurbitacin T and hundreds of derivatives have been reported. Among them, cucurbitacins B (CucB), D, E, and I have been reported to possess strong anticancer activities, while cucurbitacins F, O, P, and Q have moderate anticancer activity (Sun et al., 2010). Cucurbitacin D (CucD), which can be isolated from TK, was shown to induce apoptosis in human hepatocellular carcinoma cells (Takahashi et al., 2009) and autophagy in human T-cell leukemia cells (Nakanishi et al., 2016). It was also found to interfere with the viability of breast cancer cells (Ku et al., 2018). The anti-tumor activity of CucB has been studied for several decades as well (Hunsakunachai et al., 2019).

In our previous study, we demonstrated the antinociceptive effect of TK on paclitaxel-induced allodynia (Lee et al., 2022) and showed that spinal noradrenergic receptors play an important role in the effect of TK. Furthermore, as cucurbitacins are major components of TK, we have suggested that they may play an important role in the antinociceptive effect. However, the effect of cucurbitacins has not yet been studied.

Thus, the primary aim of this study was to assess the antinociceptive effect of two different types of cucurbitacins (CucB and CucD) in mice with paclitaxel-induced pain. The secondary aim was to assess their cytotoxic effect on cancerous and non-cancerous cells. Finally, we aimed to clarify their underlying mechanism of action on mice with paclitaxel-induced neuropathic pain.

## 2 Materials and methods

### 2.1 Collection and preparation of the plant

TK used in this experiment was produced by Hanpoong Pharm & Foods Co., Ltd., Jeonju, South Korea. The collected mature TK was cleaned and dried before storage. TK was extracted *via* the reflux method with 70% ethanol for 3 h at 100°C. The extracts were filtered and concentrated under decompression at 60°C. The required drug concentrations were prepared by diluting in the phosphate-buffered saline (PBS) at a concentration of 50 mg/ml.

### 2.2 Animals

We used six male C57BL mice (8 weeks old) in most of the experiments; only the anti-tumor growth assessment experiment was conducted on BALB mice (8 weeks old). Both kinds of animals were purchased from Dae Han Bio Link (Chungbuk, South Korea). They were placed in a specific pathogen-free animal center and housed in cages under standard experimental conditions (23°C ± 2°C; 65 ± 5% humidity; 12-h light and 12-h dark cycle). They were maintained on a standard diet (Purina, Seongnam, South Korea) with freely available water. All of the experimental protocols were approved by the Kyung Hee University Animal Care and Use Committee (approval no.: KHUASP-21-543) and were in accordance with the guidelines of the International Association for the Study of Pain.

### 2.3 Tissue collection

The mice used in immunohistochemistry (IHC), quantitative real-time polymerase chain reaction (qPCR), and high-performance liquid chromatography (HPLC) were anesthetized *via* the inhalation of isoflurane to the point of unresponsiveness. They were then transcardially perfused with cold PBS with a pH of 7.2 until the exiting blood ran clear. Brain tissue was dissected in 4% paraformaldehyde (PFA) for IHC. The mice used for qPCR or HPLC were also scarified *via* transcardial perfusion under isoflurane anesthesia without PFA fixation. Tissues were frozen and stored at −80°C until further processing.

### 2.4 Paclitaxel administration

**FIGURE 1:**
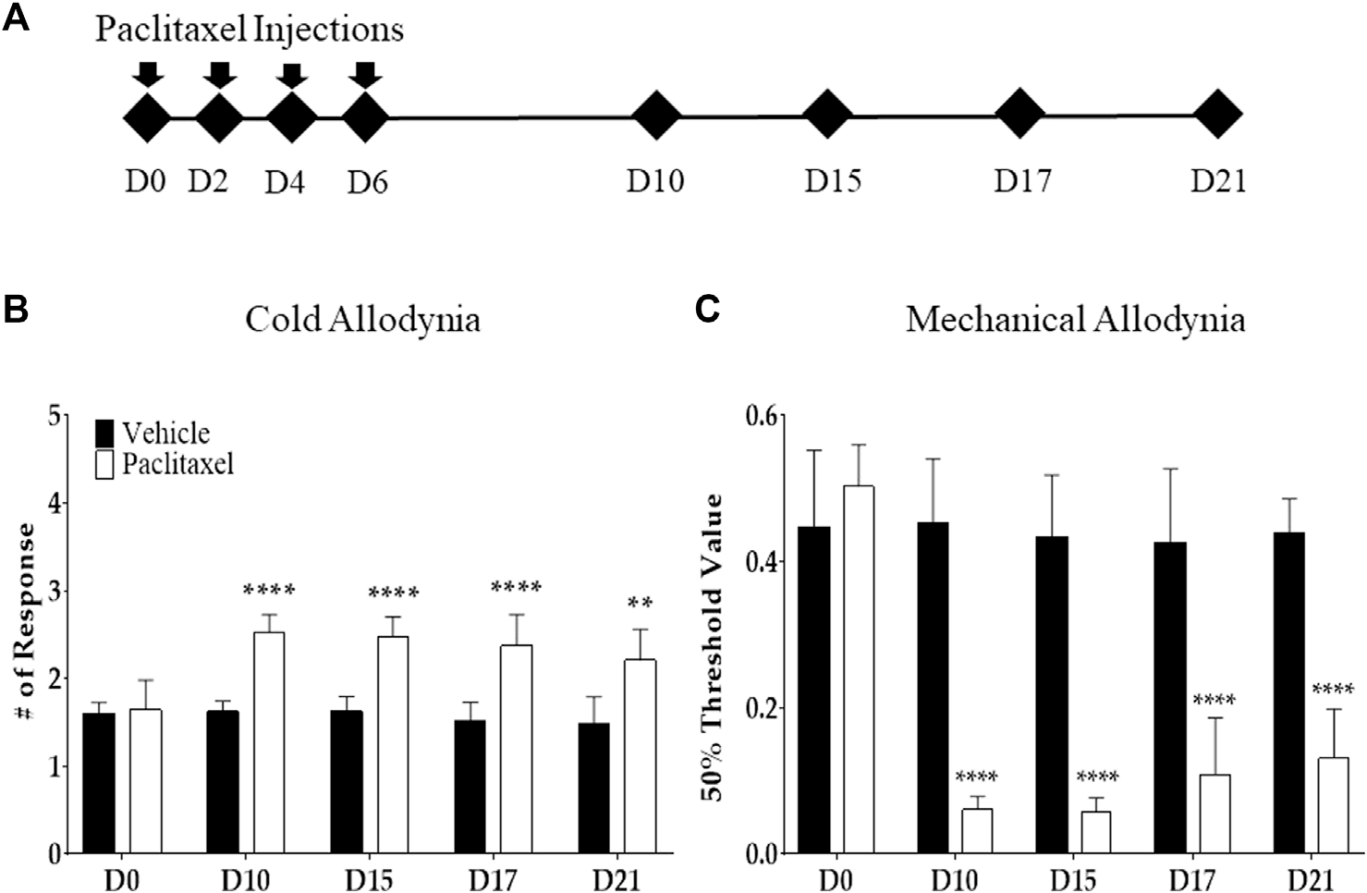
Effects of multiple paclitaxel injections on mice. Behavioral tests to assess allodynia were performed before (time point zero) and after four i.p. injections of 2 mg/kg of paclitaxel (days 0, 2, 4, and 6; an accumulative dose of 8 mg/kg). The timeline of behavioral tests **(A)** and the behavioral responses to cold **(B)** and mechanical **(C)** stimuli. Cold and mechanical stimuli were provided *via* the acetone drop and von Frey filament tests, respectively. A mixed solution of Cremophor EL and ethanol (1:1) was used as the vehicle. *N* = 8 in each group. ** *p* < .01 and **** *p* < .0001 *vs.* control with two-way ANOVA, followed by Tukey’s post-test for multiple comparisons.

Paclitaxel (Sigma Aldrich, Mo, United States) was formulated in 50% Cremophor EL and 50% absolute ethanol to a concentration of 6 mg/ml. Paclitaxel was diluted in PBS to make a final concentration of 0.2 mg/ml. The control group received the same volume of the mixture of Cremophor EL solution (Sigma Aldrich, Mo, United States) and ethanol (1:1) with a PBS dilution. The mice received an i. p. injection of paclitaxel and vehicle at a concentration of 2 mg/kg every other day (days 0, 2, 4, and 6). In total, 8 mg/kg of paclitaxel was injected to induce allodynia (Figure 1A).

### 2.5 Behavioral tests

Cold and mechanical allodynia were measured and quantified using the acetone drop and von Frey filament tests, respectively. The mice were habituated to the arena for 30 min prior to testing. Von Frey filaments (Stoelting, WI, United States) of different forces (.02–2 g) were then used to determine the 50% withdrawal threshold, as previously described (Lee et al., 2021). Briefly, the filaments were applied perpendicular to the plantar surface of the filament for up to a maximum of 5 s or until licking, flinching, or withdrawal of the paw occurred. According to Dixon’s up-down method and Chaplan’s calculation method, a 50% threshold value was calculated (Chaplan et al., 1994). The results are presented as the average of both hind paws. The acetone drop test was used to measure the response to an innocuous cold stimulus. An acetone drop (10 µL) was applied to the mid-plantar hind paw of the mice. The response latency and withdrawal frequency (number of responses) within 30 s were recorded. A positive response was considered as a lick, flinch, or withdrawal of the hind paw. Thus, the term “# of Responses” mentioned in the figures represents the average number of responses to a 10-µL acetone drop. This was conducted three times, with an interval of 5 min between applications; the total individual withdrawal durations were then calculated.

### 2.6 Cucurbitacin and noradrenaline analysis by HPLC

HPLC analysis was performed using an Agilent 1260 Infinity ll HPLC and UV detector. Cucurbitacin analysis conditions are shown in Supplementary Table S1. Stock solutions from CucB (160 μg/ml) and CucD (125 μg/ml) were prepared in methanol. The CucB and CucD used in this study were purchased from Sigma (Mo, United States). Serial dilutions of CucB (160, 80, 40, 20, 10, and 5 μg/ml) and CucD (125, 62.5, 31.25, 15.625, 7.8125, and 3.90625 μg/ml) were prepared and subjected to HPLC analysis. The standard solution was stored at −20°C in the dark until use. A sample (100 mg) was ultrasonically extracted (4°C, 30 min) with 1 ml of 30% ethanol. The diluted solution was centrifuged at 4°C at 10,000 rpm for 5 min, and the supernatant was filtered through a 0.45-µm syringe filter to obtain a test solution.

NE analysis conditions are also shown in Supplementary Table S2. The stock solution from NE (160 μg/ml) was prepared in distilled water. The NE used in this study was purchased from Sigma (Mo, United States). A total of serial dilutions of NE (50, 25, 12.5, 10, and 6.25 μg/ml) were prepared and subjected to HPLC analysis. The standard solution was stored at −20°C in the dark until use. The subsequent procedure was the same as that of the cucurbitacin analysis.

### 2.7 The effect of CucB and CucD on RAW 264.7 and Caco-2 cell lines measured by MTT proliferation assay

Cytotoxic studies were determined in RAW 264.7 and Caco-2 cell lines obtained from the Korea Cell Line Bank (ATCC). Caco-2 cells were grown in Eagle’s minimum essential medium (MEM) supplemented with 10% fetal bovine serum (FBS), and RAW 264.7 cells were grown in Dulbecco’s modified Eagle’s medium (DMEM) supplemented with 10% FBS. Cultures were maintained in a humidified atmosphere with 5% CO_2_ at 37°C and passaged bi-weekly. The viability of cells was determined *via* an MTT assay. Briefly, 1 × 10^4^ cells, suspended in 200 µL of growth medium, were seeded in 96-well plates for 24 h. After incubation, each concentration of cucurbitacin previously dissolved with dimethyl sulfoxide (DMSO) was treated for 24 h. To assess the viability, the medium was exchanged for an MTT working solution (5 mg/ml in a cell culture medium) and incubated for 1 h at 37°C. Afterward, the reaction was terminated. The formed formazan was dissolved with DMSO and measured at 490 nm using a microplate reader. The results are presented as a percentage of the control (untreated cells).

### 2.8 Quantitative real-time polymerase chain reaction

Total RNA was extracted using the AccuPrep Universal RNA extraction kit (Bioneer, Daedeok-gu, Korea), according to the manufacturer’s protocols. The concentration of RNA was quantified using a NanoDrop ND-1000 Spectrophotometer (Thermo Scientific, DE, United States). cDNA was then prepared with a Maxime RT PreMix Kit (iNtRON Biotechnology, Seongnam, South Korea). qPCR was performed using the SensiFAST SYBR No-ROX kit (Bioline, London, UK) and CFX Connect Real-time PCR Detection System (Bio-Rad, CA, United States). The oligonucleotide primers used for the PCR were as follows: *Gapdh* forward 5′-GGA GGT AGC TCC TGA TTC GC-3 and reverse 5′-CAC ATT GGG GGT AGG AAC AC-3’; α1-adrenergic receptor (*Adra1a*) forward 5′-ATG CTC CAG CCA AGA GTT CA-3′ and reverse 5′-TCC AAG AAG AGC TGG CCT TC-3’; α2-adrenergic receptor (*Adra2a*) forward 5′-AAA CCT CTT CCT GGT GTC TC-3’; 5-hydroxytryptamine receptor 1A (*Htr1a*) forward 5′-AAC TAT CTC ATC GGC TCC TT-3′ and reverse 5′-GAT TGC CCA GTA CCT GTC TA-3’; and 5-hydroxytryptamine receptor 3A (*Htr3a*) forward 5′-TGG TCC TAG ACA GAA TAG GG-3′ and reverse 5′-GGT CTT CTC CAA GTC CTG A-3’. The reaction was preheated for 10 min at 94°C, followed by 40 cycles at 94°C for 20 s, 57°C for 20 s, and 72°C for 30 s. GAPDH primers were used to standardize the amount of RNA in each sample. The reaction without cDNA was used as a negative control. We needed qPCR data for amplification-generated fluorescence to reach a specific threshold of detection (Ct value). The relative gene expression was quantified on the basis of equal amounts of RNA (.1 μg) and average Ct value for each gene. Delta Ct (ΔCt = Ct_target gene_—Ct_reference gene_) was calculated using Ct values for genes in the same sample. Actin was used as the internal reference control gene. The ΔΔCt value was obtained *via* the equation ΔΔCt = (Δ Ct_target gene_—Δ Ct_untreated_). The normalized expression fold was expressed as the value of 2^−ΔΔCT^ (*Gapdh* control = 1).

### 2.9 Immunohistochemistry

Animals were anesthetized with isoflurane, and the tissues were fixed *via* transcardial perfusion with 4% PFA. After perfusion, the tissues were collected and post-fixed with 4% PFA overnight. Following fixation, tissues were transferred to a 30% sucrose solution in PBS for 3 days at 4°C. Serial sections 10-μm thick were collected on adhesive glass slides (Mas, Japan). The slides were washed thrice for 5 min in PBS, followed by a 5-h soak in .2% Triton X-100/PBS for permeabilization. The slides were again washed in PBS, followed by a 30-min soak in 3% BAS/PBST (0.05%) for blocking. After blocking, the tissues were incubated for 24 h at 4°C in .1% BSA/PBS containing mouse anti-tyrosine hydroxylase (TH, 1:500, Novus Biologicals, United States). The slides were again washed thrice in PBS and incubated for 2 h at room temperature with Alexa Fluor 488-conjugated goat anti-rabbit IgG (H + L) (1:1,000, Abcam, United States). The slides were mounted with Vecta-shield containing DAPI and analyzed under a confocal microscope (LSM 780, Carl-Zeiss) by ZEN image software.

### 2.10 Assessment of tumor growth in tumor-injected mice

CT-26 cells (1.5 × 10^5^ cells) were subcutaneously implanted in the right flank of BALB/c mice. We selected the CT-26 cell line-bearing mouse model as it had been used in some previous studies to assess the anti-tumor effect (Yamamoto et al., 2017; Miyagi et al., 2019). The tumor weight did not exceed 10% of the body weight until the end of the experiment. The mice were treated with paclitaxel (8 mg/kg i. p., once a week for 3 w) and CucD (.5 mg/kg i. p., three times a week for 3 weeks). The measurements of tumor volumes were performed on days 7, 10, 13, 16, 19, 22, and 25. The tumor volumes were calculated as follows: volume (mm^3^) = ½ (length x width^2^). Each experiment was performed on six animals per group.

### 2.11 Statistical analysis

The statistical analyses were performed by GraphPad Prism (version 7.0. GraphPad Software Inc., United States). The data are displayed as mean ± SD. Data were first evaluated for Gaussian distribution by using the D'Agostino and Pearson normality test. Two-group comparisons were performed using the two-tailed Student’s *t*-test for unpaired samples. Statistical analyses were performed using one-way ANOVA, followed by Tukey’s test for the cell viability, gene expression, TH protein, and NE level analysis. The parametric two-way ANOVA with Sidak’s post-test for multiple comparisons was used for behavioral experiments. A level of *p* < .05 was considered significant.

## 3 Results

### 3.1 Multiple paclitaxel injections induce cold and mechanical allodynia in mice

Cold and mechanical allodynia were induced after multiple i. p. injections of paclitaxel (a cumulative dose of 8 mg/kg) in mice. The acetone drop and von Frey filament tests were used to assess each allodynia type. Behavioral tests were conducted on the first day (D0), and days 10 (D10), 15 (D15), 17 (D17), and 21 (D21), following the initial injection (Figure 1A) of paclitaxel. From D10 to D21, in the mice injected with paclitaxel, the response to cold stimuli significantly increased (Figure 1B), and the withdrawal threshold of the mechanical stimuli significantly decreased (Figure 1C). All of the significant differences between the paclitaxel-injected and control groups were <.0001. Based on these results, the consequent behavioral experiment was performed between D10 and D21, when both allodynia types were significantly developed and maintained.

### 3.2 Anti-allodynic effect of the hydroalcoholic extract of TK and CucB and CucD

**FIGURE 2:**
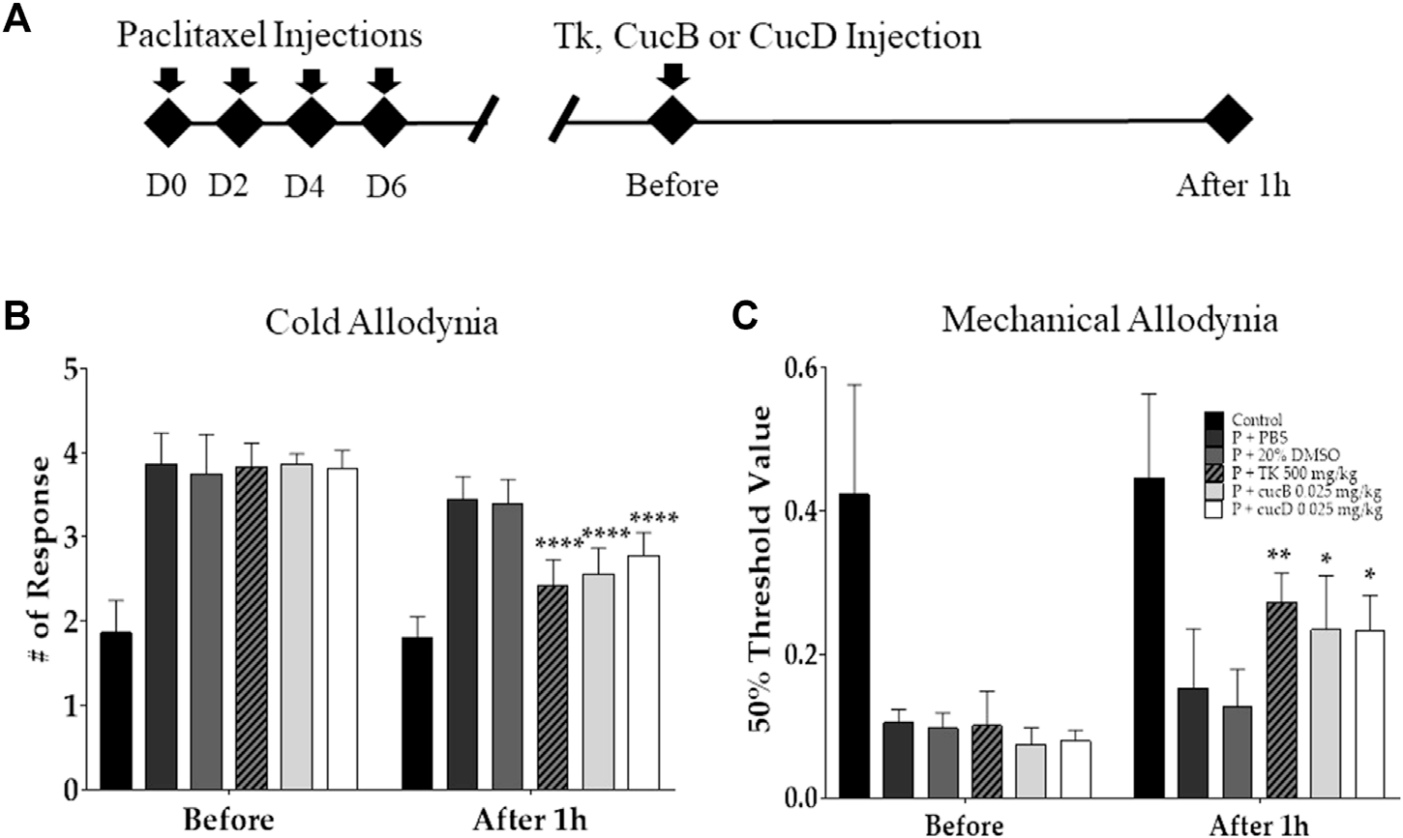
Effects of *Trichosanthes kirilowii* Maximowicz (TK) and its sub-component cucurbitacins B (CucB) and D (CucD) on cold and mechanical allodynia induced by multiple paclitaxel injections in mice. The experimental schedule **(A)** and the effects of TK, CucB, and CucD against paclitaxel-induced cold **(B)** and mechanical **(C)** allodynia in mice. Behavioral tests to measure allodynia were performed before and 1 h after the i.p. injection of PBS, 20% DMSO, 500 mg/kg of TK, .025 mg/kg of CucB, or .025 mg/kg of CucD in paclitaxel-injected mice. PBS and 20% DMSO were used as the control as they are solvents of TK and cucurbitacins, respectively. TK, *Trichosanthes kirilowii* Maximowicz; CucB, cucurbitacin B; CucD, cucurbitacin D; DMSO, dimethyl sulfoxide; P, paclitaxel. *N* = 6 in each group. * *p* < .05, ** *p* < .01, and **** *p* < .0001 *vs.* control with two-way ANOVA, followed by Tukey’s post-test for multiple comparisons.

To assess the antinociceptive effect of TK and understand the role of CucB and CucD in the effect of TK, behavioral tests were conducted after TK, CucB, and CucD treatment in mice with paclitaxel-induced neuropathic pain (Figure 2A). TK was administered orally, whereas CucB and CucD were injected i. p. CucB and CucD are known to be present in TK, and the CucB and CucD doses were calculated accordingly (Zhang et al., 2019). Multiple paclitaxel injections induced cold and mechanical allodynia, and the intensities of both allodynia types were measured before and after the administration of 500 mg/kg of TK and .025 mg/kg of CucB and of CucD. PBS and 20% DMSO were used as solvents for TK and of CucB and CucD, respectively. The results showed that TK, CucB, and CucD significantly alleviated cold and mechanical allodynia (Figures 2B,C). In the evaluation of cold allodynia, *p-*values were <.0001 at 500 mg/kg of TK, and <.001 at .025 mg/kg of CucB and .025 mg/kg of CucD. In addition, the mechanical allodynia *p-*values were .0051, .0118, and .0126, respectively, *versus* the 20% DMSO group.

### 3.3 Identification and quantification of CucB and CucD in TK

**FIGURE 3:**
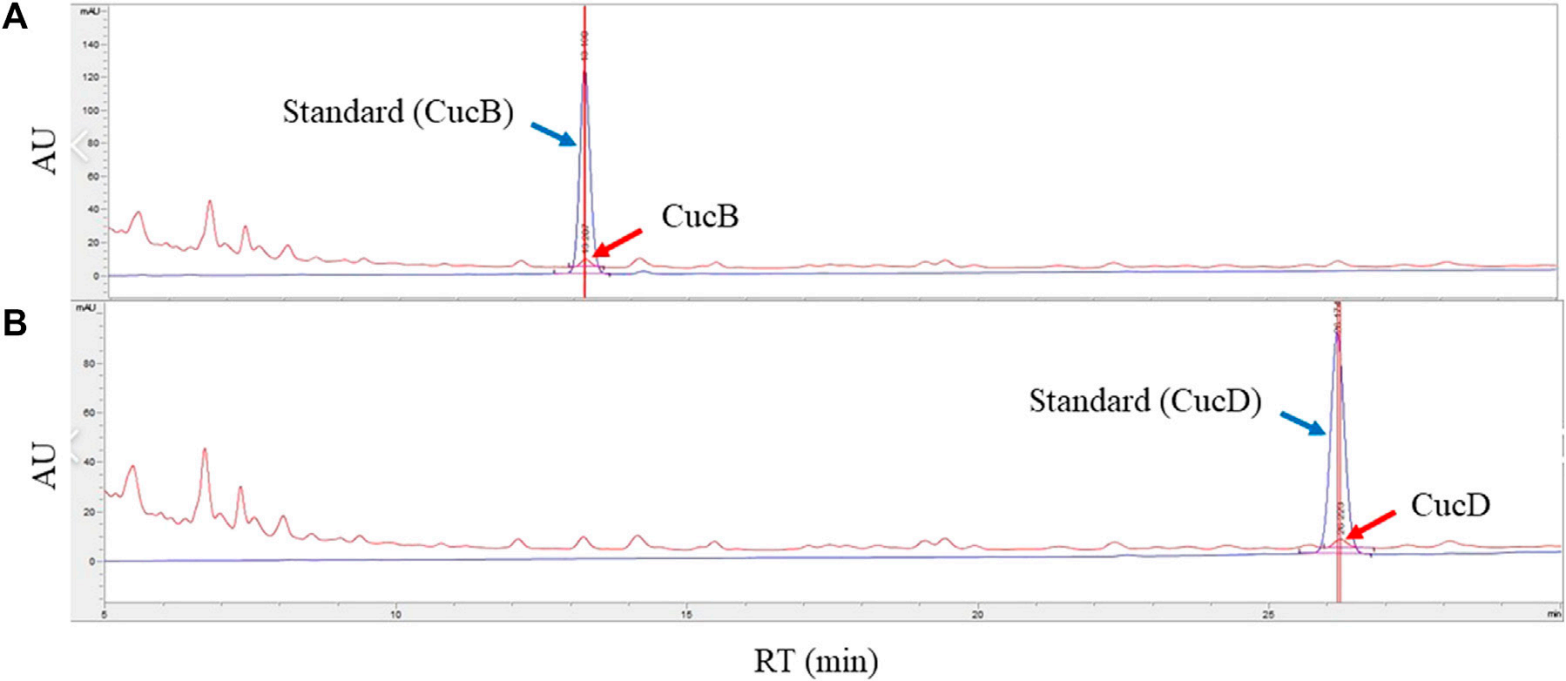
Identification and quantification of cucurbitacin B (CucB) and cucurbitacin D (CucD) in an ethanol extract of *Trichosanthes kirilowii* Maximowicz by high-performance liquid chromatography (HPLC). HPLC chromatograms of CucB **(A)** and CucD **(B)**. Peaks of the blue line (blue arrow) represent the standard, and the red line (red arrow), the CucB and CucD. The *X*-axis reports the retention time, and the *Y*-axis, the absorbance unit.

To identify CucB and CucD as active components in TK, HPLC was conducted (Figure 3). The retention time of CucB was approximately 13.2 min (Figure 3A) and that of CucD (Figure 3B) was approximately 26.2 min. The retention time and spectrum (absorbance unit) of the standard solution (red arrow) and cucurbitacin solutions (blue arrow) were consistent. Calibration curves showed the linearity of the detector over the ranges of CucB (160, 80, 40, 20, 10, and 5 μg/ml) and CucD (125, 62.5, 31.25, 15.625, 7.8125, and 3.90625 μg/ml). The CucB regression equation was y = 9.57667x + 2.93812 and RSQ = .99999, and the CucD regression equation was y = 12.67383x + 1.55528 and RSQ = .99999. The content of CucB and CucD in TK was approximately 0.0068% and 0.0048%, respectively.

### 3.4 Assessment of cell viability of CucB and CucD in RAW 264.7 and Caco-2 cells

**FIGURE 4:**
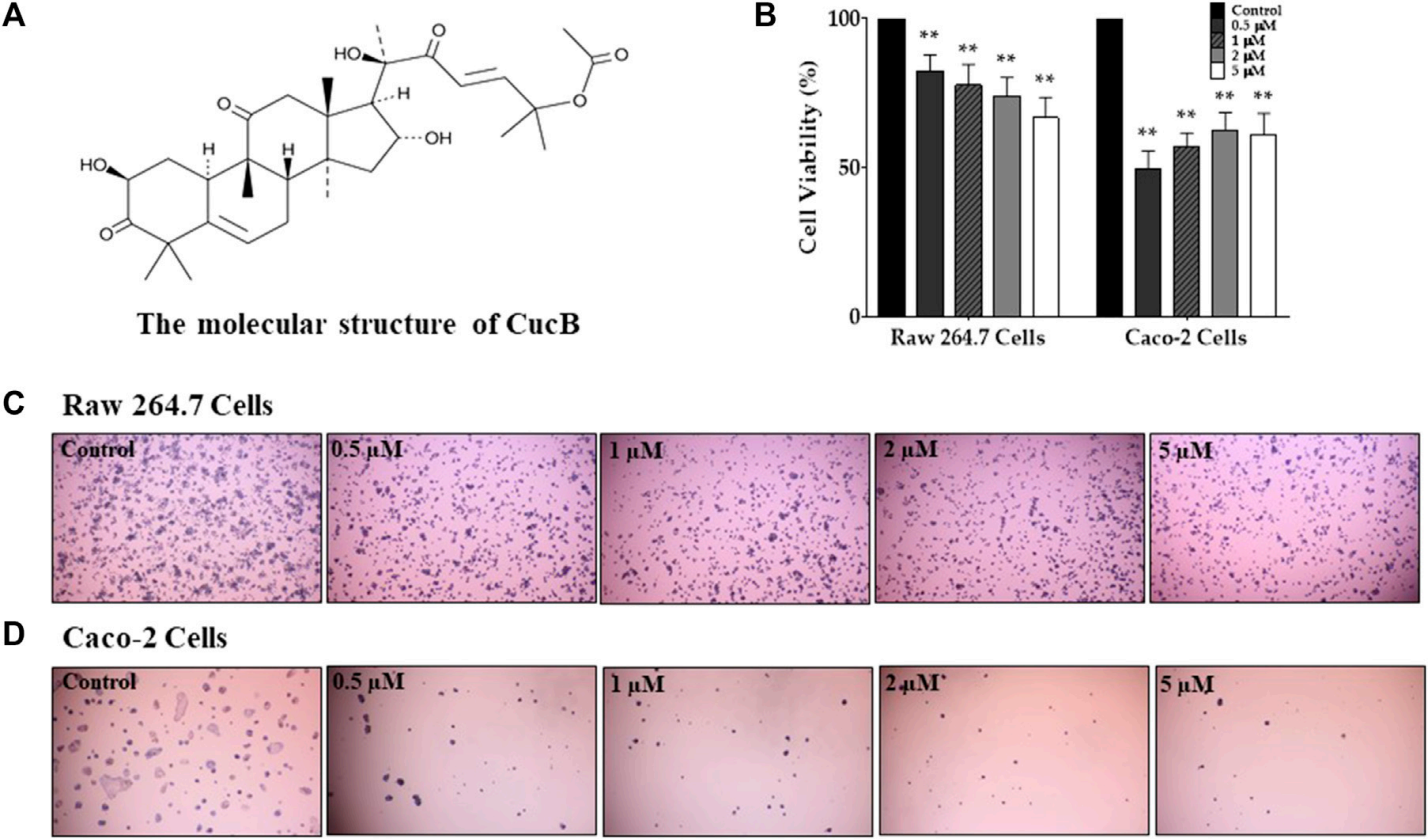
Assessment of the cytotoxic effect of cucurbitacin B (CucB). The chemical structure of CucB **(A)**. The effect of four different doses of CucB on the viability of RAW 264.7 and Caco-2 cells **(B)** and their morphologies **(C and D)**. Data were experiments and were expressed as the mean value ± SD of three independent experiments. ** *p* < .01 *vs.* control with one-way ANOVA.

**FIGURE 5:**
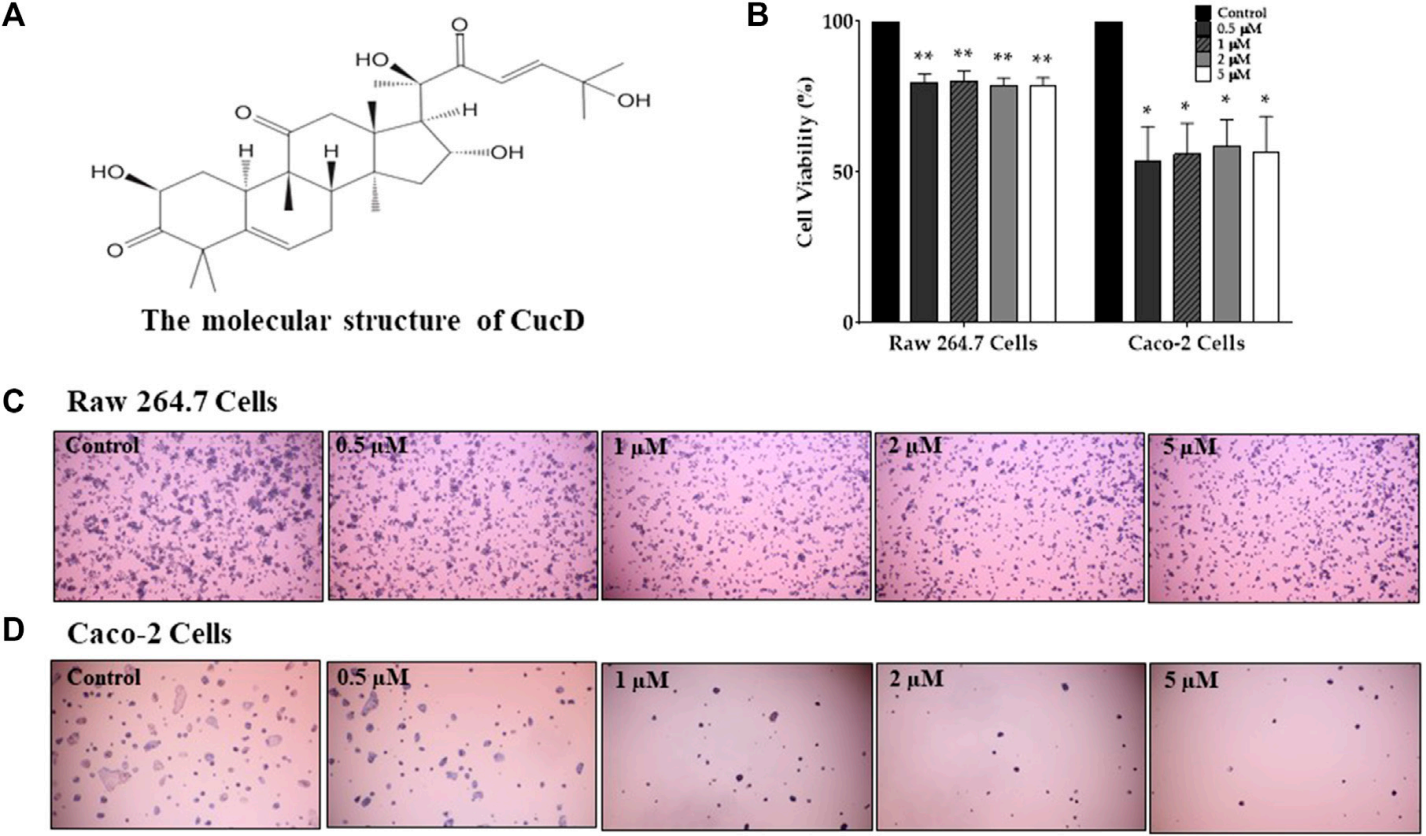
Assessment of the cytotoxic effect of cucurbitacin D (CucD). The chemical structure of CucD **(A)**. The effect of four different doses of CucD (.5, 1, 2, and 5 µM) on the viability of RAW 264.7 and Caco-2 cells **(B)** and their morphologies **(C and D)**. Data were experiments and expressed as the mean value ± SD of three independent experiments. * *p* < .05 and ** *p* < .01 *vs.* control with one-way ANOVA.

To assess the cytotoxic effect of CucB and CucD, *in vitro* experiments were conducted by using non-cancerous RAW 264.7 cells and human colonic carcinoma Caco-2 cells. The cytotoxic effects of CucB and CucD on the viability of the RAW 264.7 and Caco-2 cells are presented as percentage cell viabilities of three dependent experiments (Figures 4, 5). CucB (RAW 264.7 cell: *p-*values = 0.0045, 0.005, 0.0019, and 0.0011; Caco-2 cell: *p-*values = .0062, .0024, .004, and .007, respectively) and CucD (RAW 264.7 cell: *p-*values = .003, 0.005, .0011, and .002; Caco-2 cell: *p-*values = .0157, .0194, 0.0282, and 0.0298, respectively) showed a significant cytotoxic effect on Caco-2 cells in a concentration range between .5 µM and 5 µM. However, although CucD consistently exhibited a viability of approximately 79.3% in the concentration ranges in RAW 264.7 cells, CucB showed a dose-dependent viability as the viability decreased to 66.6% at the highest concentration (i.e., 5 µM). As these results showed that CucB has a dose-dependent cytotoxic effect in non-cancer cells (i.e., RAW 264.7), we focused on CucD in our subsequent experiments.

### 3.5 Administration of CucD alleviates paclitaxel-induced cold and mechanical allodynia dose-dependently in mice

**FIGURE 6:**
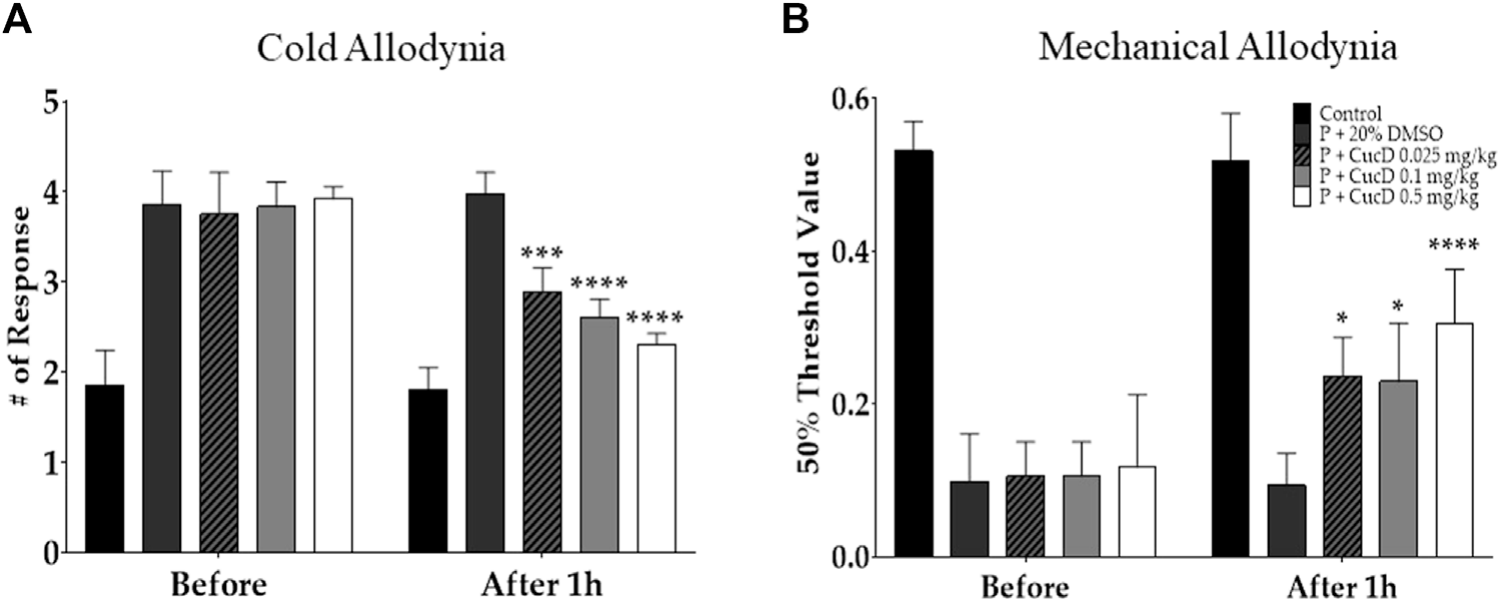
Dose-dependent effect of cucurbitacin D (CucD) on cold and mechanical allodynia induced by multiple paclitaxel injections in mice. Behavioral tests were performed twice: before (before) and 1 h after (after 1 h) the i. p. injection of 20% DMSO or of .025, .1, or .5 mg/kg of CucD in paclitaxel-injected mice. Experiments were conducted 15 days after the first paclitaxel injection. Cold **(A)** and mechanical **(B)** allodynia assessments were made *via* the acetone drop test and von Frey filament assay, respectively. P, paclitaxel; CucD, cucurbitacin D; DMSO, dimethyl sulfoxide. *N* = 7 in each group. * *p* < .05, *** *p* < .001, and **** *p* < .0001 vs. before with two-way ANOVA, followed by Tukey’s post-test for multiple comparisons.

To elucidate whether CucD has a dose-dependent antinociceptive effect on paclitaxel-induced neuropathic pain, three different doses (.025, .1, and .5 mg/kg) of CucD were administered i. p. to mice. Behavioral tests were performed before and 1 h after the CucD administrations in the allodynia-induced mice. The results show that all three doses of CucD could significantly attenuate paclitaxel-induced cold (Figure 6A) and mechanical (Figure 6B) allodynia. The effect was dose-dependent as the .5 mg/kg CucD group showed the highest anti-allodynic effect. In the evaluation of cold allodynia, *p-*values were <.0001 at .1 and 0.5 mg/kg of CucD and .0034 at .025 mg/kg of CucD compared to the 20% DMSO group. In addition, in mechanical allodynia, *p-*values were .016, .03, and <.0001, respectively, compared to the 20% DMSO group. In this study, the effect of CucD on paclitaxel-induced neuropathic pain was investigated in females compared to males. As a result of evaluating the efficacy of CucD in females, the effect was similar to that in males, and there was no difference between females and males (Supplementary Figure S1).

### 3.6 Involvement of spinal noradrenergic but not serotonergic receptors in the antinociceptive effect of CucD

**FIGURE 7:**
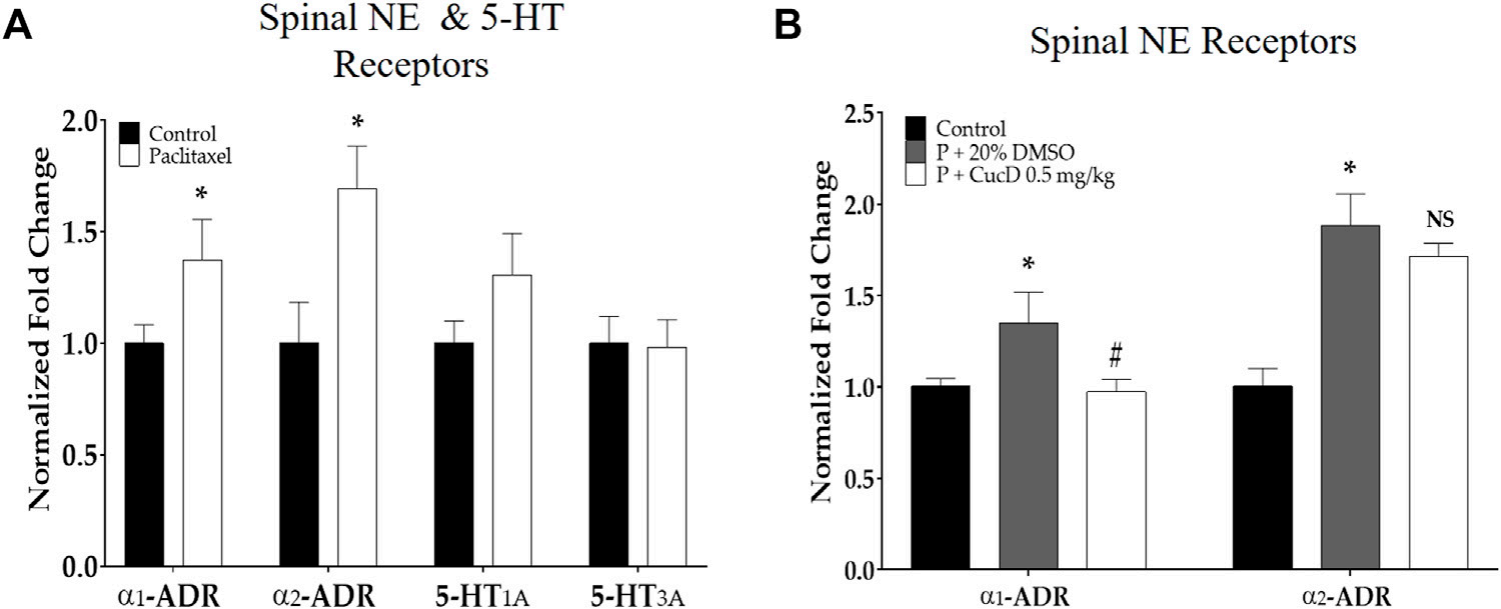
Quantitative real-time polymerase chain reaction of adrenergic and serotonergic receptor genes in the spinal cord. The effect of paclitaxel on spinal *α*_1_- and *α*_2_-adrenergic and 5-HT_1A_ and _3A_ receptors **(A)**. The effect of i p. cucurbitacin D injection on spinal *α*_1_- and *α*_2_-adrenergic receptors on mice with paclitaxel-induced neuropathic pain **(B)**. *α*_1_-ADR, *α*_1_-adrenergic receptor; *α*_2_-ADR, *α*_2_-adrenergic receptor; 5-HT_1A,_ 5-hydroxytryptamine 1A receptor; 5-HT_3A,_ 5-hydroxytryptamine 3A receptor. *N =* 6 in each group. Data were experiments and expressed as the mean value ± SD. * *p* < .05 vs. control and # *p* < .05 *vs.* P + 20% DMSO with two-tailed Student’s *t*-test A and one-way ANOVA, followed by Tukey’s post-test for multiple comparisons **(B)**.

To understand the underlying mechanism of antinociceptive action after the administration of CucD, the gene expression of noradrenergic and serotonergic receptors was evaluated from the mouse spinal cord tissue harvested 1 hour after CucD treatment. First, qRT-PCR was performed to determine the effect of paclitaxel on the expression of spinal *α*_1_- and *α*_2_-adrenergic receptors and serotonin (5-HT)_1A_ and 5-HT_3A_ receptors in the spinal cord. The results showed that paclitaxel increased the expression of *α*_1_- and *α*_2_-adrenergic receptors but not 5-HT_1A_ and 5-HT_3A_ receptors compared to the control group (Figure 7A). In a subsequent experiment, to verify whether the increased expression of adrenergic receptors is downregulated after CucD treatment, the spinal gene expression of mice treated with .5 mg/kg of CucD was analyzed (Figure 7B). The result showed that the spinal expression of *α*_1_- but not of *α*_2_-adrenergic receptors was significantly decreased compared to the paclitaxel administration group (*p-*values: .0231 and .2005, respectively). These results showed that CucD can mediate its antinociceptive effect *via* spinal *α*_1_-adrenergic receptors.

### 3.7 Tyrosine hydroxylase production in the brain and noradrenaline content in the spinal cord

**FIGURE 8:**
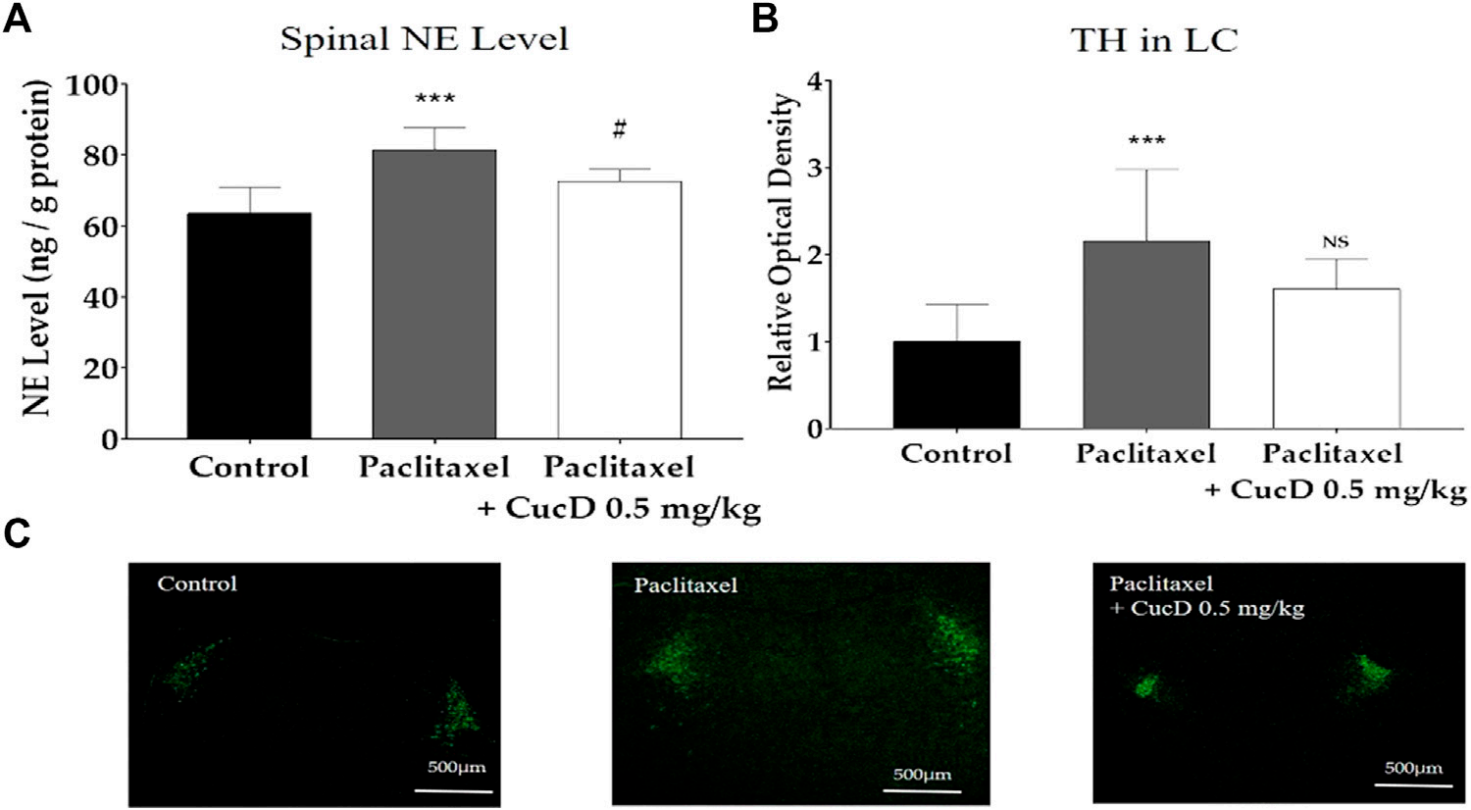
Expression levels of tyrosine hydroxylase (TH) protein immunofluorescence in mouse brains and noradrenaline content in the spinal cord. Brain sections were fixed and stained with the anti-TH antibody. The administration of paclitaxel and cucurbitacin D changed the noradrenaline level in the mouse spinal cord **(A)**. Relative intensity of TH protein production in mouse brains **(B)** and the image is representative of the slides of each group **(C)**. *N = 6* in each group. *** *p* < .001 vs. control and ^#^ *p* < .05 vs. paclitaxel with one-way ANOVA, followed by Tukey’s post-test for multiple comparisons.

To measure the NE level in the mouse spinal cord, the spinal segments of L4–5 of the mice were obtained to undergo HPLC (Figure 8A). Similar to the results of IHC conducted in the brain slice, the level of NE in the spinal cord significantly increased after paclitaxel treatment (63.61 ± 7.29 ng/g vs. 81.47 ± 6.24 ng/g; *p-*values: .0003). However, the spinal NE level after CucD administration significantly decreased to 77.67 ± 3.32 ng/g (*p*-value: .0498).

As most of the NE in the spinal cord is synthesized in the locus coeruleus (LC) of the brain, the production of TH was evaluated 1 hour after CucD administration in the brain *via* confocal microscopy (Figure 8). The staining shows the TH protein in the LC part of the brain of each group (Figure 8C). When measuring the fluorescence intensity, the value of the naïve group was established as 1 ± .42, and the paclitaxel-induced group significantly increased to 2.16 ± .83 (*p*-value: .0004). The intensity of the group treated with paclitaxel and CucD decreased to 1.6 ± .34; however, unlike the results obtained from the spinal cord, the difference was not statistically significant (*p*-value: .0851; Figure 8B).

### 3.8 Anticancer activity of CucD

**FIGURE 9:**
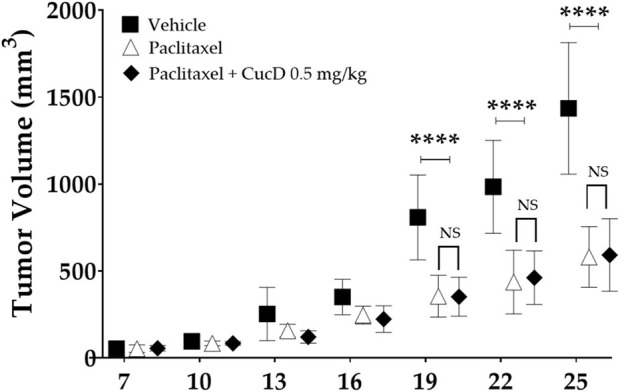
Impact of cucurbitacin D (CucD) on the anti-tumor effect of paclitaxel. CT-26 (the murine colorectal carcinoma cell line) cell-implanted mice were treated with paclitaxel (8 mg/kg, i. p.) once a week and CucD (0.5 mg/kg, i. p.), three times a week for 3 weeks. The tumor volumes were calculated as follows: volume (mm^3^) = ½ (length x width^2^). Tumor growth was expressed as the mean tumor volume ± SD of independent experiments. *N = 6* in each group and **** *p* < .0001 vs. paclitaxel and paclitaxel + CucD .5 mg/kg with two-way ANOVA, followed by Tukey’s post-test for multiple comparisons.

To assess the effect of CucD on the anti-tumor effect of paclitaxel, the anti-tumor efficacy of paclitaxel after CucD administration was investigated after subcutaneous injections of CT-26 (the murine colorectal carcinoma cell line) cells in the right flank of BALB/c mice (Figure 9). The paclitaxel treatment significantly inhibited an increase in the tumor volume compared to vehicle treatment. Results showed that the co-treatment of paclitaxel with CucD did not decrease or inhibit the anti-tumor activity of paclitaxel. Paclitaxel single or co-treatment with CucD reduced the tumor volume by 59.6% and 58.8%, respectively, on day 2,525 (*p-*values: .0168, .0393, .009, and .0063 on days 16, 19, 22, and 25, respectively, compared to the vehicle group).

## 4 Discussion

In this study, the dose-dependent antinociceptive effect of CucD against paclitaxel-induced cold and mechanical allodynia was demonstrated, along with its underlying mechanism of action. In our previous study, we demonstrated that TK could significantly decrease paclitaxel-induced allodynia and suggested that these effects may be due to CucD (Lee et al., 2022). In this study, CucD was identified in TK by HPLC along with CucB, and their toxicities on non-cancerous and cancerous cells, and RAW 264.7 and Caco-2 cells, respectively, were analyzed. Although the results showed that both CucB and CucD have similar antinociceptive effects, subsequent experiments were focused on the effect of CucD as CucB tends to have a more cytotoxic effect on non-cancerous cells than CucD (Figures 4, 5).

As the underlying mechanism of action, the involvement of the spinal NE system was demonstrated. Multiple paclitaxel injections induced an increase in spinal *α*_1_- and *α*_2_-adrenergic receptors and NE. Furthermore, it upregulated the intensity of TH-positive cells in the LC, which is the A6 noradrenergic group of neurons. The role of *α*_1_- and *α*_2_-adrenergic receptors in neuropathic pain has been reported in many papers (Martins et al., 2013; Martins et al., 2015), and the i. p. injection of both *α*_1_- and *α*_2_-adrenergic receptor antagonists was demonstrated to significantly attenuate allodynia in mice with nerve injury-induced neuropathic pain, suggesting that both receptors are involved in pain enhancement (Hord et al., 2001). In our study, the increased gene expression of *α*_1_- but not *α*_2_-adrenergic receptors was significantly downregulated 1 hour after CucD administration. Although it is difficult to determine whether the protein expression of spinal adrenergic receptors was also changed after CucD treatment, as in our study we did not conduct protein assays, several reports (Puhl et al., 2016; Kong et al., 2018) have shown that the increase and decrease in noradrenergic receptor gene expression were clearly related to the changes in protein levels when assessed by Western blot. Thus, these results suggest the protein expression of adrenergic receptors may also have been altered, following paclitaxel and CucD administration.

The LC is the primary source of NE in the brain and projects NE to diverse parts of the brain and spinal cord (Poe et al., 2020). TH is a rate-limiting enzyme in the biosynthesis of NE in the LC, and this regulatory mechanism of the TH reaction is generally considered to play a key role in controlling the catecholaminergic action (Nagatsu et al., 1964; Kobayashi et al., 2000). Whether NE in the LC inhibits or facilitates pain is still unknown, although some studies found that activating the LC resulted in pain maintenance and facilitation. Direct lidocaine administration into the LC in hyperalgesia-developed mice resulted in the inhibition of all neuropathic signs, showing that LC activation may be related to pain (Brightwell and Taylor, 2009). Furthermore, in a nerve injury-induced animal model of pain, the responsiveness of LC neurons to noxious mechanical stimuli was found to be enhanced (Viisanen and Pertovaara, 2007).

In our study, a small amount (.025 mg/kg) of CucB and CucD significantly attenuated paclitaxel-induced neuropathic pain. Furthermore, CucD showed a dose-dependent effect, and .5 mg/kg had the strongest antinociceptive effect among the three treated doses (i.e., .025, .1, and .5 mg/kg). CucD belongs to the tetracyclic triterpenes and exhibits efficacy and several characteristic mechanisms. Triterpenes are widely distributed natural compounds and are generally classified as acyclic, tetracyclic, and pentacyclic. In previous studies, tetracyclic triterpene has been reported to improve type I diabetes by inhibiting the inflammatory response that promotes the destruction of many beta cells (Kim and Kim, 2008; Keller et al., 2011). Additionally, patients’ immunological homeostasis was shown to be ameliorated after treatment (Hong et al., 2012). Furthermore, CucD directly reduced HIV replication in the central nervous system (CNS) that had HIV-infected macrophages in an *in vitro* blood–brain barrier model (Kodidela et al., 2021), suggesting that CucD could penetrate the blood–brain barrier.

The i. p. injection of .5 mg/kg of CucD tended to inhibit TH production in the LC, although the extent of inhibition was not significant. However, the spinal NE level and *α*_1_-adrenergic receptor were shown to decrease significantly. Results from our and other laboratories demonstrated that the antinociceptive effect of chemotherapy-induced neuropathic pain could be attenuated by central descending pain suppression systems, such as serotonergic and noradrenergic pathway activation (Lee et al., 2016; Cheng et al., 2019). However, in our study, multiple paclitaxel injections significantly increased the function of the noradrenergic system as spinal adrenergic receptors, and TH in the LC part of the brainstem was upregulated. Although it is difficult to fully understand the differences in the results, the function of the NE system, especially NE synthesized in the LC, may play a major role in the transition from acute to chronic pain (Taylor and Westlund, 2017).

Thus, the days when most of the experiments were conducted in our study (i.e., D15, following the initiation of paclitaxel injection) may correspond to this period, where a change in the role of the NE system occurs in paclitaxel-injected mice. This has also been suggested as the reason why, in some pain states, duloxetine, a widely known serotonin–NE reuptake inhibitor, has limited effects. In rodents, serotonin–NE reuptake inhibitors have been demonstrated to show a stronger effect at first, which decreases with the prolongation of pain (Llorca-Torralba et al., 2016). However, further studies that focus on the relation of the NE system and chemotherapy-induced neuropathic pain should be conducted to completely elucidate its role in pain.

In conclusion, our results show that CucD could be considered an option to treat paclitaxel-induced neuropathic pain as it significantly attenuated the pain and did not inhibit the anti-tumor effect of paclitaxel. For future research, standardization studies, such as whether the content of CucD is kept constant during the TK supply and demand stage and the extraction process stage, are needed. Furthermore, there should be a focus on how CucD decreases the NE level and the function of *α*_1_adrenergic receptors.).

## Data Availability

The original contributions presented in the study are included in the article/Supplementary Material; further inquiries can be directed to the corresponding authors.

## References

[B1] Beijers A. Jongen J. Vreugdenhil G. (2012). Chemotherapy-induced neurotoxicity: The value of neuroprotective strategies. Neth J. Med. 70, 18–25. 22271810

[B2] Bernabeu E. Cagel M. Lagomarsino E. Moretton M. Chiappetta D. A. (2017). Paclitaxel: What has been done and the challenges remain ahead. Int. J. Pharm. 526, 474–495. 10.1016/j.ijpharm.2017.05.016 28501439

[B3] Brightwell J. J. Taylor B. K. (2009). Noradrenergic neurons in the locus coeruleus contribute to neuropathic pain. Neuroscience 160, 174–185. 10.1016/j.neuroscience.2009.02.023 19223010 PMC2677992

[B4] Chaplan S. R. Bach F. W. Pogrel J. Chung J. Yaksh T. (1994). Quantitative assessment of tactile allodynia in the rat paw. J. Neurosci. methods 53, 55–63. 10.1016/0165-0270(94)90144-9 7990513

[B5] Chaudhry V. Rowinsky E. K. Sartorius S. E. Donehower R. C. Cornblath D. R. (1994). Peripheral neuropathy from taxol and cisplatin combination chemotherapy: Clinical and electrophysiological studies. Ann. Neurology Official J. Am. Neurological Assoc. Child Neurology Soc. 35, 304–311. 10.1002/ana.410350310 7907208

[B6] Chen F. Wang C. C. Kim E. Harrison L. E. (2008). Hyperthermia in combination with oxidative stress induces autophagic cell death in HT‐29 colon cancer cells. Cell Biol. Int. 32, 715–723. 10.1016/j.cellbi.2008.02.010 18396422

[B7] Chen J. C. Chiu M. H. Nie R. L. Cordell G. A. Qiu S. X. (2005). Cucurbitacins and cucurbitane glycosides: Structures and biological activities. Nat. Product. Rep. 22, 386–399. 10.1039/b418841c 16010347

[B8] Cheng Z. Zhang M. Ling C. Zhu Y. Ren H. Hong C. (2019). Neuroprotective effects of ginsenosides against cerebral ischemia. Molecules 24, 1102. 10.3390/molecules24061102 30897756 PMC6471240

[B9] Durand J. Deplanque G. Montheil V. Gornet J. Scotte F. Mir O. (2012). Efficacy of venlafaxine for the prevention and relief of oxaliplatin-induced acute neurotoxicity: Results of EFFOX, a randomized, double-blind, placebo-controlled phase III trial. Ann. Oncol. 23, 200–205. 10.1093/annonc/mdr045 21427067

[B10] Dworkin R. H. O’connor A. B. Backonja M. Farrar J. T. Finnerup N. B. Jensen T. S. (2007). Pharmacologic management of neuropathic pain: Evidence-based recommendations. Pain 132, 237–251. 10.1016/j.pain.2007.08.033 17920770

[B11] Farshchian N. Alavi A. Heydarheydari S. Moradian N. (2018). Comparative study of the effects of venlafaxine and duloxetine on chemotherapy-induced peripheral neuropathy. Cancer Chemother. Pharmacol. 82, 787–793. 10.1007/s00280-018-3664-y 30105459

[B12] Finnerup N. B. Attal N. Haroutounian S. Mcnicol E. Baron R. Dworkin R. H. (2015). Pharmacotherapy for neuropathic pain in adults: A systematic review and meta-analysis. Lancet Neurol. 14, 162–173. 10.1016/S1474-4422(14)70251-0 25575710 PMC4493167

[B13] Hong Y. J. Kim N. Lee K. Sonn C. H. Lee J. E. Kim S. T. (2012). Korean red ginseng (Panax ginseng) ameliorates type 1 diabetes and restores immune cell compartments. J. Ethnopharmacol. 144, 225–233. 10.1016/j.jep.2012.08.009 22925946

[B14] Hord A. H. Denson D. D. Stowe B. Haygood R. M. (2001). alpha-1 and alpha-2 Adrenergic antagonists relieve thermal hyperalgesia in experimental mononeuropathy from chronic constriction injury. Anesth. Analgesia 92, 1558–1562. 10.1097/00000539-200106000-00042 11375846

[B15] Hunsakunachai N. Nuengchamnong N. Jiratchariyakul W. Kummalue T. Khemawoot P. (2019). Pharmacokinetics of cucurbitacin B from *Trichosanthes cucumerina* L. in rats. BMC complementary Altern. Med. 19, 157–168. 10.1186/s12906-019-2568-7 PMC6609384 31272429

[B16] Jayaprakasam B. Seeram N. P. Nair M. G. (2003). Anticancer and antiinflammatory activities of cucurbitacins from Cucurbita andreana. Cancer Lett. 189, 11–16. 10.1016/s0304-3835(02)00497-4 12445672

[B17] Jordan M. (2002). Mechanism of action of antitumor drugs that interact with microtubules and tubulin. Curr. Med. Chemistry-Anti-Cancer Agents 2, 1–17. 10.2174/1568011023354290 12678749

[B18] Kaushik U. Aeri V. Mir S. R. (2015). Cucurbitacins–an insight into medicinal leads from nature. Pharmacogn. Rev. 9, 12–18. 10.4103/0973-7847.156314 26009687 PMC4441156

[B19] Keller A. C. Ma J. Kavalier A. He K. Brillantes A.-M. B. Kennelly E. J. (2011). Saponins from the traditional medicinal plant Momordica charantia stimulate insulin secretion *in vitro* . Phytomedicine 19, 32–37. 10.1016/j.phymed.2011.06.019 22133295 PMC3389550

[B20] Kim K. Kim H. Y. (2008). Korean red ginseng stimulates insulin release from isolated rat pancreatic islets. J. Ethnopharmacol. 120, 190–195. 10.1016/j.jep.2008.08.006 18773949

[B21] Kobayashi K. Noda Y. Matsushita N. Nishii K. Sawada H. Nagatsu T. (2000). Modest neuropsychological deficits caused by reduced noradrenaline metabolism in mice heterozygous for a mutated tyrosine hydroxylase gene. J. Neurosci. 20, 2418–2426. 10.1523/JNEUROSCI.20-06-02418.2000 10704516 PMC6772502

[B22] Kodidela S. Sinha N. Kumar A. Kumar S. (2021). Anti-HIV activity of cucurbitacin-D against cigarette smoke condensate-induced HIV replication in the U1 macrophages. Viruses 13, 1004. 10.3390/v13061004 34072078 PMC8228815

[B23] Kong W. N. Cui Y. Fu Y. J. Lei Y. Ci Y. Bao Y. (2018). The α1‐adrenergic receptor is involved in hepcidin upregulation induced by adrenaline and norepinephrine via the STAT3 pathway. J. Cell. Biochem. 119, 5517–5527. 10.1002/jcb.26715 29377263

[B24] Kosturakis A. K. He Z. Li Y. Boyette-Davis J. A. Shah N. Thomas S. K. (2014). Subclinical peripheral neuropathy in patients with multiple myeloma before chemotherapy is correlated with decreased fingertip innervation density. J. Clin. Oncol. 32, 3156–3162. 10.1200/JCO.2013.54.5418 25154818 PMC4171359

[B25] Ku J. M. Hong S. H. Kim H. I. Lim Y. S. Lee S. J. Kim M. (2018). Cucurbitacin D exhibits its anti-cancer effect in human breast cancer cells by inhibiting Stat3 and Akt signaling. Eur. J. Inflamm. 16, 1721727X1775180. 10.1177/1721727x17751809

[B26] Kuroi K. Shimozuma K. (2004). Neurotoxicity of taxanes: Symptoms and quality of life assessment. Breast cancer 11, 92–99. 10.1007/BF02968010 14718800

[B27] Lee J. H. Go D. Kim W. Lee G. Bae H. Quan F. S. (2016). Involvement of spinal muscarinic and serotonergic receptors in the anti-allodynic effect of electroacupuncture in rats with oxaliplatin-induced neuropathic pain. Korean J. physiology Pharmacol. official J. Korean Physiological Soc. Korean Soc. Pharmacol. 20, 407–414. 10.4196/kjpp.2016.20.4.407 PMC4930909 27382357

[B28] Lee J. H. Ji H. Ko S.-G. Kim W. (2021). Ji017 attenuates oxaliplatin-induced cold allodynia via spinal trpv1 and astrocytes inhibition in mice. Int. J. Mol. Sci. 22, 8811. 10.3390/ijms22168811 34445514 PMC8396301

[B29] Lee J. H. Kim B. Ko S.-G. Kim W. (2022). Analgesic effect of SH003 and Trichosanthes kirilowii Maximowicz in paclitaxel-induced neuropathic pain in mice. Curr. Issues Mol. Biol. 44, 718–730. 10.3390/cimb44020050 35723335 PMC8929024

[B30] Llorca-Torralba M. Borges G. Neto F. Mico J. A. Berrocoso E. (2016). Noradrenergic Locus Coeruleus pathways in pain modulation. Neuroscience 338, 93–113. 10.1016/j.neuroscience.2016.05.057 27267247

[B31] Martins I. Carvalho P. De Vries M. G. Teixeira-Pinto A. Wilson S. P. Westerink B. H. (2015). Increased noradrenergic neurotransmission to a pain facilitatory area of the brain is implicated in facilitation of chronic pain. Anesthesiology 123, 642–653. 10.1097/ALN.0000000000000749 26146901

[B32] Martins I. De Vries M. Teixeira-Pinto A. Fadel J. Wilson S. Westerink B. (2013). Noradrenaline increases pain facilitation from the brain during inflammatory pain. Neuropharmacology 71, 299–307. 10.1016/j.neuropharm.2013.04.007 23602988

[B33] Miyagi A. Kawashiri T. Shimizu S. Shigematsu N. Kobayashi D. Shimazoe T. (2019). Dimethyl fumarate attenuates oxaliplatin-induced peripheral neuropathy without affecting the anti-tumor activity of oxaliplatin in rodents. Biol. Pharm. Bull. 42, 638–644. 10.1248/bpb.b18-00855 30930422

[B34] Nagatsu T. Levitt M. Udenfriend S. (1964). Tyrosine hydroxylase: The initial step in norepinephrine biosynthesis. J. Biol. Chem. 239, 2910–2917. 10.1016/s0021-9258(18)93832-9 14216443

[B35] Nakanishi T. Song Y. He C. Wang D. Morita K. Tsukada J. (2016). Autophagy is associated with cucurbitacin D-induced apoptosis in human T cell leukemia cells. Med. Oncol. 33, 30–38. 10.1007/s12032-016-0743-y 26913856

[B36] Pachman D. R. Barton D. L. Watson J. C. Loprinzi C. L. (2011). Chemotherapy-induced peripheral neuropathy: Prevention and treatment. Clin. Pharmacol. Ther. 90, 377–387. 10.1038/clpt.2011.115 21814197

[B37] Poe G. R. Foote S. Eschenko O. Johansen J. P. Bouret S. Aston-Jones G. (2020). Locus coeruleus: A new look at the blue spot. Nat. Rev. Neurosci. 21, 644–659. 10.1038/s41583-020-0360-9 32943779 PMC8991985

[B38] Puhl S. L. Kazakov A. Müller A. Fries P. Wagner D. Böhm M. (2016). Adenosine A1 receptor activation attenuates cardiac hypertrophy and fibrosis in response to α1‐adrenoceptor stimulation *in vivo* . Br. J. Pharmacol. 173, 88–102. 10.1111/bph.13339 26406609 PMC4813379

[B39] Ríos J. L. Escandell J. M. Recio M. C. (2005). New insights into the bioactivity of cucurbitacins. Stud. Nat. Prod. Chem. 32, 429–469.

[B40] Rowinsky E. K. Chaudhry V. Cornblath D. R. Donehower R. C. (1993). Neurotoxicity of taxol. J. Nat. Cancer Inst. Monogr. 15, 107–115. 7912516

[B41] Rowinsky E. K. Donehower R. C. (1995). Paclitaxel (taxol). N. Engl. J. Med. 332, 1004–1014. 10.1056/NEJM199504133321507 7885406

[B42] Stillman M. Cata J. P. (2006). Management of chemotherapy-induced peripheral neuropathy. Curr. Pain Headache Rep. 10, 279–287. 10.1007/s11916-006-0033-z 16834943

[B43] Sun C. Zhang M. Shan X. Zhou X. Yang J. Wang Y. (2010). Inhibitory effect of cucurbitacin E on pancreatic cancer cells growth via STAT3 signaling. J. cancer Res. Clin. Oncol. 136, 603–610. 10.1007/s00432-009-0698-x 19816711 PMC11828316

[B44] Takahashi N. Yoshida Y. Sugiura T. Matsuno K. Fujino A. Yamashita U. (2009). Cucurbitacin D isolated from Trichosanthes kirilowii induces apoptosis in human hepatocellular carcinoma cells *in vitro* . Int. Immunopharmacol. 9, 508–513. 10.1016/j.intimp.2009.01.006 19185617

[B45] Taylor B. K. Westlund K. N. (2017). The noradrenergic locus coeruleus as a chronic pain generator. J. Neurosci. Res. 95, 1336–1346. 10.1002/jnr.23956 27685982 PMC5374049

[B46] Viisanen H. Pertovaara A. (2007). Influence of peripheral nerve injury on response properties of locus coeruleus neurons and coeruleospinal antinociception in the rat. Neuroscience 146, 1785–1794. 10.1016/j.neuroscience.2007.03.016 17445989

[B47] Yamamoto S. Ushio S. Egashira N. Kawashiri T. Mitsuyasu S. Higuchi H. (2017). Excessive spinal glutamate transmission is involved in oxaliplatin-induced mechanical allodynia: A possibility for riluzole as a prophylactic drug. Sci. Rep. 7, 9661. 10.1038/s41598-017-08891-1 28851920 PMC5574967

[B48] Zhang H.-Q. Liu P. Duan J.-A. Dong L. Shang E.-X. Qian D.-W. (2019). Hierarchical extraction and simultaneous determination of flavones and triterpenes in different parts of Trichosanthes kirilowii Maxim. by ultra-high-performance liquid chromatography coupled with tandem mass spectrometry. J. Pharm. Biomed. Analysis 167, 114–122. 10.1016/j.jpba.2019.02.003 30763882

