# Supplementary material for: The involvement of the noradrenergic system in the antinociceptive effect of cucurbitacin D on mice with paclitaxel-induced neuropathic pain
Source: Front Pharmacol. 2023 Jan 4;13:1055264. doi: 10.3389/fphar.2022.1055264 (PMC9846532; doi:10.3389/fphar.2022.1055264)
Supplement: Supplementary file 1 [file Table1.docx]

|  | | | | |
| --- | --- | --- | --- | --- |
| Treatment | Condition |  |  |  |
| Column | Fortis extended-C18  (4.6 mm x 250 mm, 5 μm) |  |  |  |
| Flow rate | 1mL/min |  |  |  |
| Injection volume | 10 μL |  |  |  |
| UV detection | 230 |  |  |  |
| Run time | 50 min |  |  |  |
|  | Time (min) | %ACN | %DW |  |
| Gradient | 0 | 55 | 45 |  |
|  | 30 | 80 | 50 |  |
|  | 35 | 100 | 0 |  |
|  | 40 | 100 | 0 |  |
|  | 45 | 55 | 45 |  |
|  | 50 | 55 | 45 |  |
|  | |  |  |  |

Table 1. Analytical conditions of HPLC for analysis of cucurbitacin
