# Supplementary figures and images for: The involvement of the noradrenergic system in the antinociceptive effect of cucurbitacin D on mice with paclitaxel-induced neuropathic pain
Source: Front Pharmacol. 2023 Jan 4;13:1055264. doi: 10.3389/fphar.2022.1055264 (PMC9846532; doi:10.3389/fphar.2022.1055264)

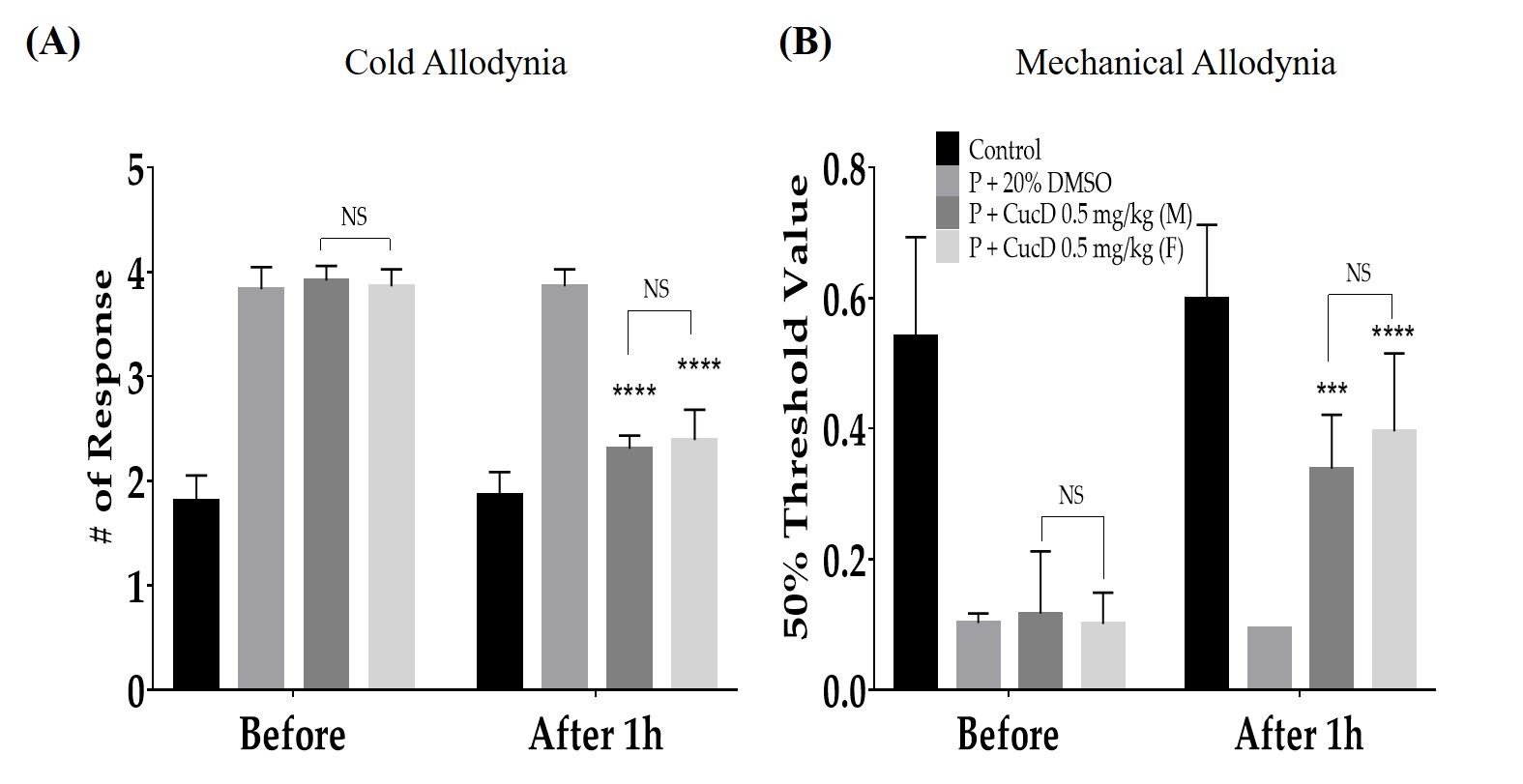

Supplement: Supplementary file 2 [file Image1.jpeg]
