# Supplementary material for: The involvement of the noradrenergic system in the antinociceptive effect of cucurbitacin D on mice with paclitaxel-induced neuropathic pain
Source: Front Pharmacol. 2023 Jan 4;13:1055264. doi: 10.3389/fphar.2022.1055264 (PMC9846532; doi:10.3389/fphar.2022.1055264)
Supplement: Supplementary file 3 [file Table2.docx]

|  | | | | |
| --- | --- | --- | --- | --- |
| Treatment | Condition |  |  |  |
| Column | Fortis Triart-C18  (4.6 mm x 250 mm, 5 μm) |  |  |  |
| Flow rate | 1mL/min |  |  |  |
| Injection Volume | 10 μL |  |  |  |
| UV detection | 220 |  |  |  |
| Run time | 40 min |  |  |  |
|  | Time (min) | %ACN | %DW |  |
| Gradient | 0 | 0 | 100 |  |
|  | 10 | 0 | 100 |  |
|  | 15 | 100 | 0 |  |
|  | 20 | 100 | 0 |  |
|  | 30 | 0 | 100 |  |
|  | 45 | 0 | 100 |  |
|  | |  |  |  |

Table 2. Analytical conditions of HPLC for analysis of NE
